# Modified method for effective primary vascular smooth muscle progenitor cell culture from peripheral blood

**DOI:** 10.1007/s10616-020-00419-2

**Published:** 2020-09-09

**Authors:** Jin-Hee Seong, Yi-Sun Song, Hyun-Woo Joo, In-Hwa Park, Guang-Yin Shen, Na-Kyoung Shin, A-Hyeon Lee, Amy M. Kwon, Yonggu Lee, Hyuck Kim, Kyung-Soo Kim

**Affiliations:** 1 Graduate School of Biomedical Science and Engineering, Hanyang University, Seoul, South Korea; 2 Division of Cardiology, Department of Internal Medicine, Hanyang University College of Medicine, Seoul, South Korea; 3 Biostatistical Consulting and Research Laboratory, Medical Research Collaborating Center, Industry-University Cooperation Foundation, Hanyang University, Seoul, South Korea; 4 Department of Internal Medicine, Hanyang University Guri Hospital, Guri, South Korea; 5 Department of Thoracic Surgery, Hanyang University Seoul Hospital, Seoul, South Korea; 6 Division of Cardiology, Department of Internal Medicine, Jilin University Jilin Central Hospital, Jilin, China

**Keywords:** VSMPCs, PBMCs, PDGF-BB, Peripheral blood, Cell culture

## Abstract

In previous studies, vascular smooth muscle progenitor cells (vSMPCs) isolated from peripheral blood mononuclear cells (PBMCs) were cultured using medium containing platelet-derived growth factor-BB (PDGF-BB) for 4 weeks. However, this method requires long culture periods of up to 4 weeks and yields low cell counts. Therefore, we proposed the modified method to improve the cell yield and purity and to reduce the cell culture period. PBMCs were isolated from human peripheral blood and cultured by the conventional method using medium containing PDGF-BB alone or the modified method using medium containing PDGF-BB, basic fibroblast growth factor (bFGF), and insulin-transferrin-selenium ITS for 4 weeks. The purity of vSMPCs was analyzed for the expression of a- smooth muscle actin (SMA) by flow cytometry and significantly higher in the modified method than conventional methods at the 1st and 2nd weeks. Also, mRNA expression of a-SMA by real-time PCR was significantly higher in the modified method than conventional method at the 2 weeks. The yield of vSMPCs by trypan blue exclusion assay was significantly higher in the modified method than conventional method at the 1st, 2nd and 3rd weeks. The primary culture using the modified method with PDGF-BB, bFGF, and ITS not only improved cell purity and yield, but also shortened the culture period, compared to the conventional culture method for vSMPCs. The modified method will be a time-saving and useful tool in various studies related to vascular pathology.

## Introduction

Vascular smooth muscle progenitor cells (vSMPCs) are defined as circulating or resident cells localized within the bone marrow (BM), vascular wall, or perivascular area that are committed to the smooth muscle fate and capable of differentiating into mature smooth muscle cells (SMCs) (Merkulova-Rainon et al. 2012). Previous reports have demonstrated the role of bone marrow (BM)-derived smooth muscle progenitor cells in contributing towards the development and progression of atherosclerotic vascular diseases and neointima hyperplasia after vascular injuries (Han et al. 2001; Iwata et al. 2010; Sata et al. 2002; Shimizu et al. 2001; Zernecke et al. 2005; Zoll et al. 2008). vSMPCs were shown to be involved in the mechanism of plaque formation in an atherosclerotic animal model, as well as the process of neointimal formation after arterial injury.

Platelet-derived growth factor-BB (PDGF-BB) is involved in various physiological functions including blood vessel formation, vascular growth, and proliferation of SMCs. It has also been known to promote vSMPC differentiate into mature SMCs (Battegay et al. 1994; Hellstrom et al. 1999). Previous reports have described that vSMPCs could be cultured from peripheral blood mononuclear cells (PBMCs) in the medium containing PDGF-BB (Kang et al. 2014; Lin et al. 2016; Simper et al. 2002; Sugiyama et al. 2006; Xie et al. 2008). However, this conventional primary culture method requires at least a 4-week-long culture period and generally yields low cells counts (Wang et al. 2012). To use vSMPCs more efficiently in vascular disease research, it is desirable to develop a method for obtaining higher yields of vSMPCs in a shorter period of time than the conventional method using PDGF-BB containing medium.

Several growth factors and supplements have been introduced to improve the result of primary vascular SMC culture from the artery walls. Basic fibroblast growth factor (bFGF) is known to be involved in angiogenesis and to promote migration and proliferation of SMCs in the presence of PDGF-BB in the medium (Jackson and Reidy 1993; Kato et al. 1998; Millette et al. 2005). Supplementation of insulin-transferrin-selenium (ITS) in the medium has also been reported to reduce the use of serum and promote cell proliferation during primary culture of aortic SMCs (Chua et al. 2005; Waldbillig and Pang 1992).

Therefore, supplementation of these factors to the culture medium during the primary culture of vSMPCs may improve the efficacy of primary SMC culture. Here, we propose a newly modified method that uses a culture medium containing PDGF-BB, bFGF and ITS for the primary culture of vSMPCs from PBMCs. The purity and abundance of the vSMPCs cultured by the modified method were compared with those cultured by the conventional method using medium containing PDGF-BB alone.

## Materials and methods

### Isolation of vascular smooth muscle progenitor cells from human peripheral blood

**Fig. 1:**
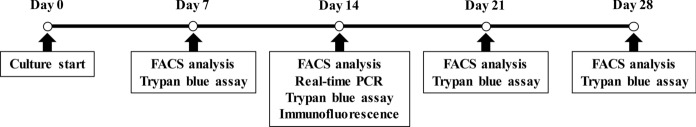
Experimental scheme of vSMPCs culture. vSMPCs was cultured in the modified method and the conventional method for 4 weeks at 1 week of intervals, respectively

The study protocol was reviewed and approved by Institutional Review Board of Hanyang University Medical Center (IRB No.; HYUH201506005003). Adult peripheral blood samples were obtained from 2 healthy human volunteers (1 male, age 29; 1 female, age 25) after informed consent was obtained. 20 ml of human peripheral blood were collected in sterile vacutainer tubes containing the anticoagulant EDTA (BD Vacutainer Systems, Roborough, Plymouth, UK). PBMCs were isolated from the buffy coat blood after density gradient centrifugation using Lymphoprep (STEMCELL Technologies Inc., Vancouver, Canada). After washing 3 times with phosphate buffered saline (PBS; Gibco, Grand Island, NY, U.S.), the PBMCs was cultured in dulbecco’s modified eagle’s medium (DMEM; Gibco, Grand Island, NY, USA) containing 20% fetal bovine serum (FBS; Gibco, Grand Island, NY, USA); 1% 100 U/ml penicillin and 100 mg/ml streptomycin (P.S; Gibco, Grand Island, NY, USA); 50 ng/ml PDGF-BB (R&D systems Inc., Minneapolis, Minnesota, U.S.) (Conventional method) or DMEM containing 20% FBS; 1% P.S; 5 ng/ml PDGF-BB; 10 ng/ml bFGF (Biobud, Seongnam-si, Gyeonggi-do, Republic of Korea); ITS (10 ng/ml insulin-5.5 ng/ml Transferrin–5 ng/ml Selenium; Gibco, Grand Island, NY, U.S.) (Modified method) and placed on 6 well plate coated with type I collagen (Corning, Corning, New York, U.S.) at a density of 2 × 106/well. The cells were maintained in a humidified 37 °C incubator with 5% CO2. The medium was replaced every 5 days. PBMCs was seeded at the 1 week intervals for 4 weeks (Fig. 1).

### Fluorescence-activated cell sorting (FACS) analysis

The FACS analysis was performed to identify the α-smooth muscle actin (SMA) expression in the vSMPCs cultured in both methods at 1st, 2nd, 3rd, and 4th week of experiment. Cells were harvested with StemPro accutase cell dissociation reagent (Gibco, Grand Island, NY, U.S.) and resuspended in 10% FBS/0.1% sodium azide in PBS. Cells were fixed in Methanol:Acetone = 1:1 for 10 min, intracellular antibodies permeabilized with 0.1% TritonT-100 (Sigma-aldrich, St. Louis, Missouri, U.S.) in PBS. The cells were incubated for 30 min with the primary antibodies to Alexa488-conjugaed human α- SMA (Abcam, Cambridge, UK) in 3% bovine serum albumin (Sigma-aldrich, St. Louis, Missouri, U.S.) in PBS. After washing in 10% FBS/0.1% sodium azide (Sigma-aldrich, St. Louis, Missouri, U.S.) with PBS 3 times, analysis was performed using a FACS Canto flow cytometer (BD Biosciences, Franklin Lakes, New Jersey, U.S.). Data were analyzed using FACSDIVA software (BD Biosciences, San jose, California, U.S.). The experiments were repeated three times.

### RNA isolation and quantitative real-time polymerase chain reaction (qRT-PCR)

qRT-PCR was performed to identify the amount of expression of a-SMA mRNA indicating the abundance of SMPCs cultured in both methods at 2 weeks of experiment. Total RNA was extracted from vSMPCs by using the Qiazol reagent (Qiagen, Hilden, Germany) following the manufacturer’s instructions. RNA concentrations were measured with a Nanodrop ND-2000 spectrophotometer (Thermo Fisher Scientific Inc., Waltham, Massachusetts, U.S.), and purity was determined by measuring ratios of A260 and A280, which ranged from 1.8 to 2.0.

For qRT-PCR, complementary DNA (cDNA) was synthesized from 3 ug of RNA using Moloney Murine Leukemia virus reverse transcriptase primed with oligo (dT) (Invitrogen, California, U.S.). The qRT-PCR was performed using a Light Cycler 480 System (Roche, Basel, Switzerland) with a FastStart DNA Master SYBR Green I kit (Roche Diagnostics, Indianapolis, U.S.). qPCR amplification was performed with an initial incubation for 10 min at 95 °C followed by 45 cycles of 10 s at 95 °C, 10 s at 60 °C,and 8 s at 72 °C and then a final dissociation step at 65 °C for 15 s. The crossing point (Cp) of each sample was automatically determined by the LightCycler program. The primers for human α-smooth muscle actin (SMA) were forward 5′-CCGAGATCTCACTG ACTACCTCATG-3′, reverse 5′-ACAATCTCACGCTCAGCAGTAGTAA-3′. The primers for human glyceraldehyde 3-phosphate dehydrogenase (GAPDH) were forward 5′-GGTATGACAACGAATTTGGCTACAG-3′, reverse 5′-TCTCTCTCTTCCTCTTGTGCTCTTG-3′. PCR was performed in duplicate and the measured transcript levels were normalized against those of GAPDH. The experiments were repeated three times.

### Measurement of cell yield

Trypan blue exclusion assay was performed to determine the yield of vSMPCs in both methods at 1st, 2nd, 3rd, and 4th week of experiment. Cells were harvested with StemPro accutase cell dissociation reagent and resuspended in PBS. Cell suspension was stained by 0.4% trypan blue dye (Gibco, Grand Island, NY, U.S.). Trypan blue-negative cells was counted with a hemocytometer (Paul Marienfeld GmbH & Co. KG, Am Wöllerspfad, Lauda-Königshofen, Germany) under an inverted light microscope (XDS-3LT, OPTIKA Italy, Via Rigla, Ponteranica BG, Italy) at 100 × magnification. The vSMPCs yield was calculated by multiplying the number of harvested cells to the percentage of a-SMA positive cells at 1, 2, 3, and 4 weeks and dividing the value by the number of PBMCs initially. The experiments were repeated three times.

$$\begin{aligned} \text{Cell yield}\,(\% )\, = \,\left( \text{Number of harvested cells}\, \times \,\alpha - \text{SMA positive cells}\, \% \right)\, \hfill \\ / \,\left( \text{Initially number of PBMCs} \right). \hfill \\ \end{aligned}$$

### Immunofluorescence (IF) staining

Immunofluorescence staining was performed to confirm the expression of α-SMA and smooth muscle myosin heavy chain (smMHC) indicating the SMCs phenotype in SMPCs cultured in both methods at 2 weeks of experiment. Cells were fixed in methanol:acetone = 1:1 for 10 min, permeabilized in 0.1% TritonX-100 for 5 min. Primary antibodies were used against α-SMA, smMHC, cluster of differentiation 31 (CD31) (Abcam, Cambridge, UK). α-SMA and CD31 was blocked in 10% normal donkey serum (Vector Laboratories Inc., Burlingame, California, U.S.) in PBS. smMHC was blocked in 10% normal goat serum (Abcam, Cambridge, UK) in PBS. The cells were incubated at 4 °C with the primary antibodies to mouse anti-α-SMA, rabbit anti-smMHC, and mouse anti-CD31. After washing in PBS, the cells were incubated for 1 h at room temperature with the secondary antibodies. Nuclei were stained with 4′,6-diamidino-2-phenylindole (DAPI; Vector Laboratories Inc., Burlingame, California, U.S.) Immunofluorescent image captured using an inverted fluorescence microscope (LeicaDMI4000B, Wetzlar, Germany) at 200 × magnification. Fluorescence-positive cells were counted in order to calculate the marker expression (number of fluorescence-labeled cells/number of total nuclei) in randomly 4 images in each group. The experiments were repeated three times.

### Statistical analysis

We performed Kruskal–Wallis tests to examine whether there are significant differences of rank-sum in α-SMA positive cell (%) and cell yields by the two different methods at four different culturing periods at α = 0.05 to avoid normal assumption due to sample size.Besides, we tested if there is a significant difference in mRNA expression levels and quantitative fluorescence analysis at the 2nd week according to Kruskal–Wallis tests. All statistical methods were conducted using SAS 9.4 (SAS institute Inc. NC, USA).

## Results

### Purity of vascular smooth muscle progenitor cells according to culture period

**Fig. 2:**
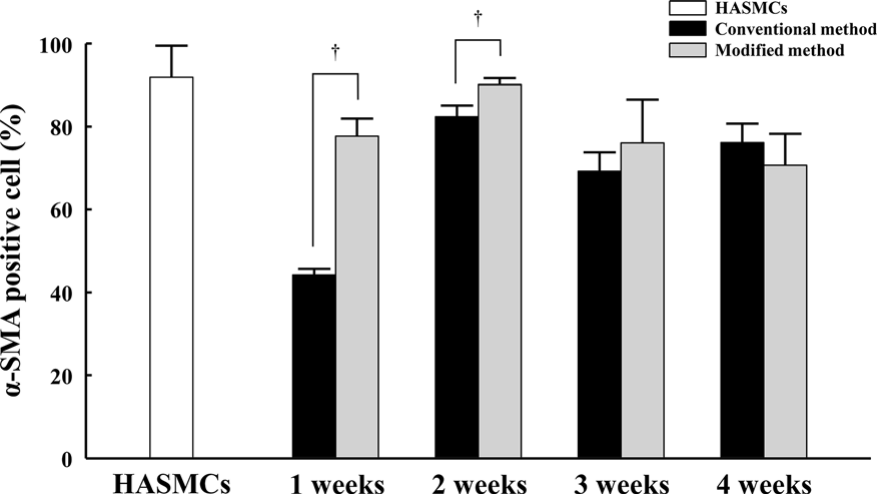
The purity of vSMPCs measured with α-SMA expression by FACS analysis. α-SMA positive cell in HASMCs were measured as a positive control. All data are expressed as mean ± SD. †*P* < 0.05, Modified method *vs.* Conventional method. The experiments were performed in triplicate

In order to compare the purity of vSMPCs according to culture period, the percentage of α-SMA positive cells was analyzed by FACS (Fig. 2 and Table 1). Population of α-SMA positive cells was significantly different between the conventional method and the modified method (*P* = 0.0353). The purity of vSMPCs was significantly higher in the modified method than that in the conventional method at 1 week (*P *= 0.0495) and 2 weeks of culture (*P* = 0.0495). The purity of vSMPCs was the highest at 2 weeks of culture in both culture method; however, the purity rapidly reached 77.7% at 1 week of culture and remained stable after 2 weeks of culture in the modified method. The purity slowly reached 44.25% at 1 week of culture and was peaked after 2 weeks of culture in the conventional method. Cell purity did not significantly different between the vSMPCs at 2 weeks of culture in both culture method and in human aortic smooth muscle cells (HASMCs) as a positive control. (Modified method vs. HASMCs, P = 0.513; Conventional method vs. HASMCs, *P* = 0.127).

**Table 1:** Kruskal-Wallis test (α-SMA positive cell (%))

Methods	χ^2^	*P* value
Conventional vs. Modified	4.45	0.0353***
Conventional vs. Modified at the 1st week	3.8571	0.0495***
Conventional vs. Modified at the 2nd week	3.8571	0.0495***
Conventional vs. Modified at the 3rd week	0.7843	0.3758
Conventional vs. Modified at the 4th week	1.1905	0.2752

χ^2^; Test statistic, ***p-value < 0.05

### mRNA expression of α-smooth muscle actin

**Fig. 3:**
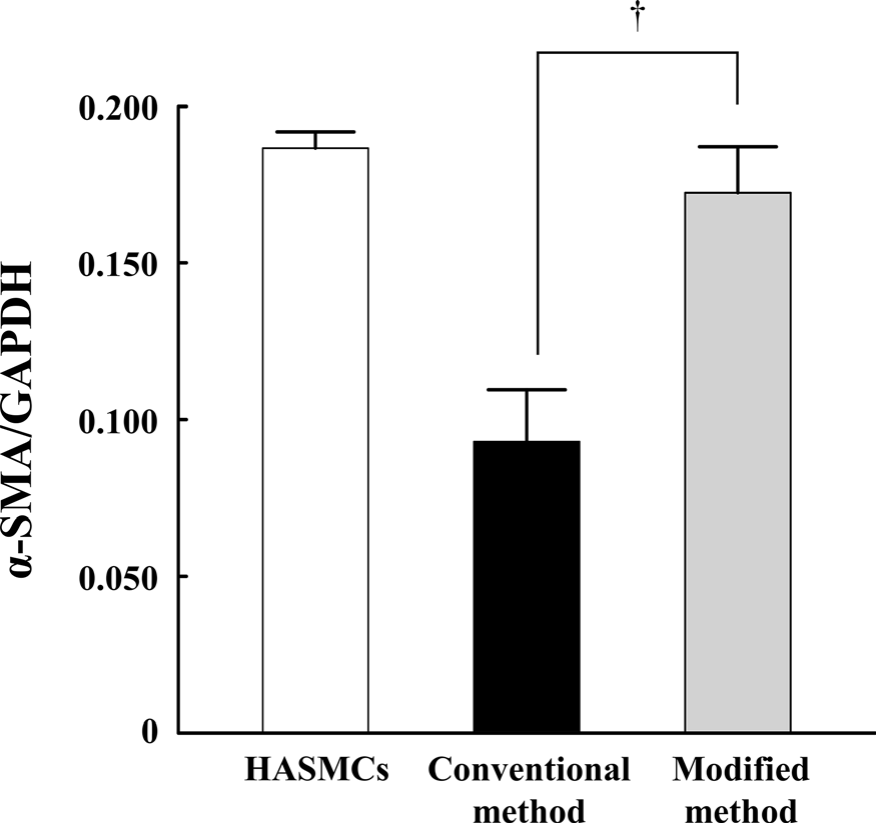
mRNA expression of α-SMA in vSMPCs at 2 weeks measured by real-time PCR. mRNA expression of α-SMA in HASMCs was measured as a positive control. The transcripts levels were normalized by comparison with GAPDH. All data are expressed as mean ± SD. †*P *< 0.05, Conventional method *vs.* Modified method. The experiments were performed in triplicate

The expression of α-SMA mRNA in vSMPCs cultured at 2 weeks was confirmed by real-time PCR (Fig. 3 and Table 2). The expression of α-SMA mRNA was significantly higher in the modified method than that in the conventional method (*P* = 0.0495). And, expression of α-SMA mRNA did not significantly different between in the modified method and in HASMCs as a positive control (*P* = 0.345), whereas was significantly lower in the conventional method than that in HASMCs (*P* = 0.046).

**Table 2:** Kruskal-Wallis Test (mRNA expression)

Methods	χ^2^	P-value
Conventional vs. Modified	3.8571	0.0495***

χ^2^; Test statistic value, ***p-value < 0.05

### Cell yield according to culture period

**Fig. 4:**
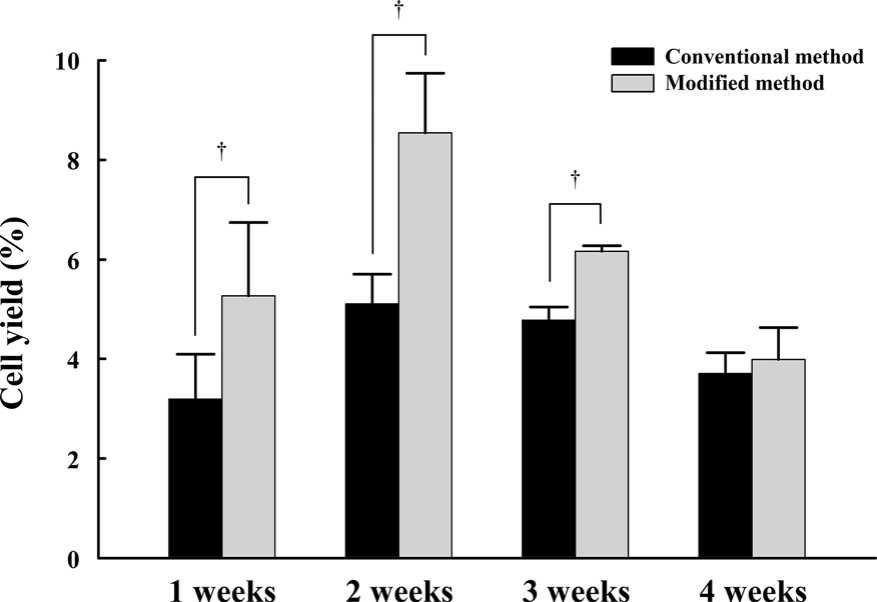
The yield of vSMPCs assessed by trypan blue exclusion assay. All data are expressed as mean ± SD. †*P* < 0.05, Modified method *vs.* Conventional method. The experiments were performed in triplicate

In order to compare the cell yield according to culture period, cell number was performed by trypan blue exclusion assay (Fig. 4 and Table 3). The cell yield was significantly different between the conventional method and the modified method (*P* = 0.0179). The cell yield was significantly higher in the modified method than that in the conventional method at 1 week (*P* = 0.0495), 2 weeks (*P *= 0.0495) and 3 weeks of culture (*P* = 0.495). The yield of cells peaked at 2 weeks of culture, then gradually decreased afterwards in both culture methods.

**Table 3:** Kruskal-Wallis test (yield)

Methods	χ^2^	P-value
Conventional vs. Modified	5.6033	0.0179***
Conventional vs. Modified at the 1st week	3.8571	0.0495***
Conventional vs. Modified at the 2nd week	3.8571	0.0495***
Conventional vs. Modified at the 3rd week	3.8571	0.0495***
Conventional vs. Modified at the 4th week	1.1905	0.2752

χ^2^; Test statistic, ***p-value < 0.05

### Morphologic appearance of vascular smooth muscle progenitor cells

**Fig. 5:**
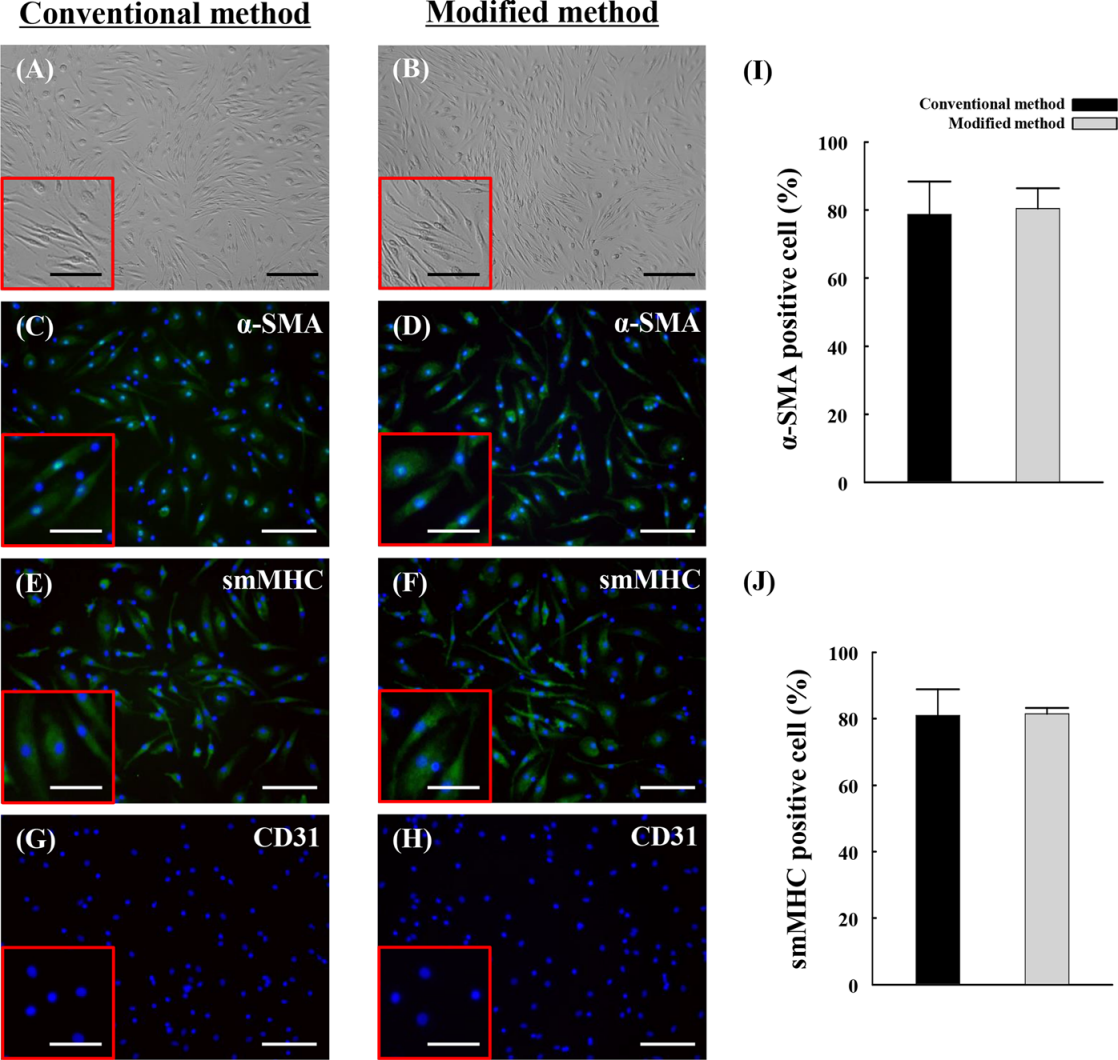
Morphologic appearance and phenotype of vSMPCs observed by microscope. (**a**, **b**) Hill-and-valley morphology. (**c**, **d**) α-SMA, (**e**, **f**) smMHC, (**g**, **h**) CD31 of immunofluorescence (green). (**i**, j) Result of quantitative analysis of smooth muscle cells specific marker α-SMA and smMHC. CD31 of endothelial surface markers were stained as a negative control. Nuclei were stained with 4′,6-diamidino-2-phenylindole (DAPI) (blue). Scale bar: 200 μm (**a**, **b**); 100 μm (**c**–**h**, in the insets of **a**, **b**); 50 μm (in the insets of **c**–**h**). The experiments were performed in triplicate

To confirm the cell morphologic appearance, vSMPCs were observed by microscopy. vSMPCs cultured in both culture methods at 2 weeks were appeared to be spindle-shaped cells with hill and valley pattern as a smooth muscle cell’s morphology (Fig. 5a, b). To additionally evaluate phenotype, cells was stained using smooth muscle cell specific antibodies. The vSMPCs cultured in both methods at 2 weeks were observed expression of smooth muscle cells specific marker α-SMA (Fig. 5c, d) and smMHC (Fig. 5e, f) by immunofluorescence staining. In the quantitative analysis results, the percentage of α-SMA and smMHC positive cells did not significantly different between in the two method at 2 weeks(α-SMA, *P* = 0.770; smMHC, *P* = 0.905) (Fig. 5i, j). Endothelial markers such as CD31 were stained to confirm the presence of endothelial cells and mononuclear cells in the cultured cells. However, expression of the endothelial cell surface marker CD31 was not observed in vSMPCs cultured in both methods at 2 weeks (Fig. 5g, h).

## Discussion

In this study, the purity and abundance of vSMPCs were compared between the conventional culture method and the modified culture method, using the medium containing PDGF-BB alone and one containing a combination of PDGF-BB, bFGF, and ITS, respectively. The vSMPCs cultured in the modified medium showed higher purity and abundance than those cultured in the conventional medium at 2 weeks of culture. The purity of vSMPCs increased more rapidly from the 1st week of culture and remained steady after 2 weeks of culture under the modified medium, compared with that under the conventional medium. The yield of vSMPCs in the modified method was determined to be approximately twice that of the conventional method at 2 weeks of culture.

vSMPCs have been identified in the circulation, bone-marrow, and vascular walls (Majesky et al. 2011; van Oostrom et al. 2009). Circulating vSMPCs were originally inferred from animal studies that demonstrated the possible recruitment of circulating BM-derived cells to sites of arterial remodeling and the ability of haematopoietic stem cells to differentiate, in vitro and in vivo, into SMCs (Merkulova-Rainon et al. 2012; Sata et al. 2002; Shimizu et al. 2001). vSMPCs in mouse models stimulate collagen synthesis and reduce infiltration of inflammatory cells, thereby promoting a stable phenotype during the development and progression of atherosclerosis (Zoll et al. 2008). A distinct lineage of neointimal cells derived from circulating bone marrow-derived cells, resembling immature vascular SMCs, is recruited during vascular repair after major vessel damage (Han et al. 2001). The expression of SDF-1α has been implicated in neointima formation by mediating mobilization and recruitment of vSMPCs from peripheral blood while the levels of SDF-1α are known to be differentially modulated in response to apoptosis after arterial injury (Schober et al. 2004???; Zernecke et al. 2005). In addition, recent studies have reported that the number of vSMPCs is higher in patients with vascular disease than in healthy individuals. Sugiuama et al. reported the presence of higher number of circulating cells expressing CD14-CD105 and a-SMA from peripheral blood in the coronary artery disease (CAD) patients compared to that in non-CAD patients (Sugiyama et al. 2006). These studies suggest that vSMPCs is closely related to the pathogenesis and repair mechanisms of various vascular diseases and injuries, implicating their wide utility in research studies associated with the field.

PDGF-BB is well-known for regulating blood vessel formation and vascular growth and promoting SMC proliferation and migration (Battegay et al. 1994; Hellstrom et al. 1999; Jawien et al. 1992; Xie et al. 2014). Zhao et al. have reported that PDGF-BB promotes human pulmonary arterial SMC proliferation and survival (Zhao et al. 2014). PDGF-BB has also been known to stimulate the differentiation of vSMPCs into SMCs (Lin et al. 2016; Simper et al. 2002; Wang et al. 2016). In light of these findings, PDGF-BB has been used as the growth factor of choice in vSMPCs primary cultures. Thus, the conventional method for vSMPCs primary culture form peripheral blood employed the culture medium containing PDGF-BB alone as growth factors.

A long period for primary culture may be an important setback for researchers employing the conventional culture method. Primary culture for vSMPC conventionally requires long culture period of up to 4 weeks, as reported in previous studies. Simper et al. reported that only six to eight vascular progenitor colonies appear from total PBMCs after 3 weeks in culture with medium containing PDGF-BB (Simper et al. 2002). Kang et al. reported that vSMPC morphology begins to appear for about 4 weeks with medium containing PDGF-BB (Kang et al. 2014). Although these studies established the significance of PDGF-BB as a critical factor for vSMPC culture, they did not assess the efficacy of culture throughout the culture periods. In this study, we assessed the purity and yield on a weekly basis and, surprisingly, found that the purity and yield of vSMPCs peaked at 2 weeks, rather than at 4 weeks of culture, as described in the previous studies. This finding may thus provide many researchers ample scope to shorten research periods significantly.

Previous studies employing the conventional medium containing 50 ng of PDGF-BB, which is 10 time higher than that in the modified medium (Joo et al. 2014; Lin et al. 2016; Simper et al. 2002). Our pilot studies have shown no morphological differences in vSMPCs cultured in the modified method when the concentration of PDGF-BB was 50 ng and 5 ng.

Wang et al. reported that the yield of vSMPCs cultured by the conventional method using medium containing PDGF-BB was lower (Wang et al. 2012). In our study, the yield of vSMPCs in presence of the conventional medium at 2 weeks was low (about 5%), whereas the yield of vSMPCs reached more than 8% in the modified method at 2 weeks of culture.

bFGF is a growth factor and signaling protein and mediates SMC migration (Chen et al. 2016b; Kato et al. 1998; Schroder et al. 2007). Several studies have reported that bFGF has the most robust effect on the ability of SMCs to induce a contractile to proliferative phenotype switch, in addition to controlling their growth and proliferation (Lindner and Reidy 1991; Shi and Chen 2014). Induction of bFGF signaling in SMCs inhibits TGFβ signaling and converts contractile SMCs to the proliferative phenotype (Chen et al. 2016a). PDGF-BB induces the release of bFGF and proliferation of human SMCs (Millette et al. 2005). Jackson et al. suggested that bFGF plays an important role in stimulating SMC migration and potentiates the movement of SMCs into the intima (Jackson and Reidy 1993). In the present study, it was anticipated that supplementation of bFGF promotes vSMPCs proliferation and provides a higher yield in the modified culture medium than in the conventional culture medium.

ITS is a medium supplement that reduces the use of serum and promotes the proliferation of many cells, including SMCs (Libby and O’Brien 1983). Chua et al. demonstrated the benefits of ITS supplementation in human chondrocytes monolayer culture (Chua et al. 2005). Lua et al. have shown that ITS may be permissive for sustained differentiation of embryonic cardiac muscle cells (Lau 1993). ITS supplemented medium in the aortic SMCs enhanced survival and prevents protein loss (Libby and O’Brien 1983). Thus, the role of ITS in the modified vSMPC culture medium may have been to sustain their differentiation into SMCs by promoting protein synthesis to express the SMC phenotype. Our results showed that vSMPCs in the modified culture medium containing PDGF-BB, bFGF, and ITS, were morphologically characterized as smooth muscle cells expressing their specific markers such as α-SMA and smMHC.

Although this study paves the way for rapid culturing of vSMPCs through PBMC primary culture, we also acknowledge certain limitations. First, the related mechanisms by which PDDG-BB, bFGF, and ITS reduced the culture period and increased the yield in the culture of vSMPCs have not been delineated. Second, the optimal concentrations for PDGF-BB, bFGF, and ITS in the modified medium are yet to be established. Further investigation is needed to decide optimal concentrations of PDGF-BB, bFGF, and ITS in the culture of vSMPCs. Third, it remains to be elucidated as to what caused the high purity and yields of vSMPCs at 2 weeks in the conventional method in this study, unlike in the previous studies in which the purity and yields peaked at 4 weeks of culture. Finally, it is necessary to study a large cohort of human peripheral blood samples in the future. The number of sample for each group was small. Therefore, future studies should involve large number of sample.

Nevertheless, in conclusion, our results suggest that our modified method for vSMPC culture from peripheral blood using a medium supplemented with PDGF-BB, bFGF, and ITS could provide higher purity and better yield of vSMPCs than the conventional culture method. In addition, the modified method shortened the culture period for vSMPCs. Overall, our improved culture method may provide a valuable resource to researchers in the field of cardiovascular diseases, especially arterial injury and repair, for conducting experiments more efficiently with vSMPCs derived from the peripheral blood.

## Abbreviations

α-SMA: Alpha-smooth muscle actin
bFGF: Basic fibroblast growth factor
BM: Bone marrow
CD31: Cluster of differentiation 31
HASMCs: Human aortic smooth muscle cells
ITS: Insulin-transferrin-selenium
PBMCs: Peripheral blood mononuclear cells
PDGF-BB: Platelet-derived growth factor-BB
SMCs: Smooth muscle cells
smMHC: Smooth muscle myosin heavy chain
vSMPCs: Vascular smooth muscle progenitor cells
